# Transient Response to Liberation Maneuvers in Central Positional Nystagmus Due to Cerebral Metastases Mimicking Benign Paroxysmal Positional Vertigo– A Case Report

**DOI:** 10.1007/s12311-025-01851-w

**Published:** 2025-05-09

**Authors:** Maritta Spiegelberg, Joao Lemos, Sun-Uk Lee, Jeong-Yoon Choi, Alexander Andrea Tarnutzer

**Affiliations:** 1 Neurology, Cantonal Hospital of Baden, Baden, 5404 Switzerland; 2 Faculty of Medicine, Coimbra University, Coimbra, Portugal; 3 Neurology Department, Coimbra University Hospital Centre, Coimbra, Portugal; 4 Neurotology and Neuro-Ophthalmology Laboratory, Korea University Medical Center, Seoul, 02841 Republic of Korea; 5 Department of Neurology, Korea University Medical Center, Seoul, 02841 Republic of Korea; 6 Dizziness Center, Clinical Neuroscience Center, Department of Neurology, Seoul National University Bundang Hospital, Seongnam, Republic of Korea; 7 Department of Neurology, Seoul National University College of Medicine, Seoul, Republic of Korea; 8 Faculty of Medicine, University of Zurich, Zurich, Switzerland

**Keywords:** Central positional nystagmus, Misdiagnosis, Supine roll test, Gufoni maneuver, Tumor

## Abstract

**Background:**

Benign paroxysmal positional vertigo (BPPV) is by far the most frequent cause of positional nystagmus (PN). However, PN may also be encountered in central lesions. In this case report we describe a patient with isolated positional vertigo and central PN which mimicked a lateral-canal cupulithiasis, including initial response to liberation maneuvers.

**Case Description:**

A 44-year-old male patient reported new-onset position-dependent vertigo with nausea and gait-imbalance for 10 days. During supine roll testing for the lateral semicircular canals, he showed a persistent apogeotropic PN (being more intense left-ear-down) accompanied by moderate vertigo. Except for the PN, the neurologic examination was normal. He received a diagnosis of a apogeotropic-variant right-lateral canal BPPV and responded well to a Gufoni maneuver (nose-up). However, on follow-up, apogeotropic PN showed-up again, converted into a geotropic variant after a Barbecue-liberation maneuver, and then disappeared. Due to the re-emergence of the initial PN on the second follow-up consult, a brain-MRI was requested, disclosing disseminated infra- and supratentorial cystic brain metastases. The largest mass compressed midline cerebellar structures. Urgent surgical resection revealed a histopathologic diagnosis of an adeno-carcinoma of the lung.

**Discussion:**

Short-lasting responses to liberation maneuvers may also be seen in CPN, mimicking the response-pattern expected in BPPV cases. Thus, response to treatment must be validated on a follow-up consultation. Pressure by a cystic cerebellar mass lesion on the nodulus and uvula that varies with changing head-position relative to gravity, resulting in adaptational changes in PN direction and intensity could explain our findings.

## Introduction

Positional vertigo (PV) accounts for 10.5–21% of patients being evaluated for vertigo or dizziness [1]. Among cases of positional vertigo, benign paroxysmal positional vertigo (BPPV) is by far the most frequent cause [2]. Depending on the affected semicircular canal (SCC), the induced vertigo is accompanied by characteristic positional nystagmus (PN), which can be paroxysmal (i.e., lasting ≤ 60 s) or persistent (i.e., lasting > 60 s), based on the location of the otoconia within the SCC (canal lumen vs. cupula). Given its prevalence and diversity in subtypes, having a thorough understanding is crucial for every frontline provider.

However, it is important to acknowledge that central lesions can mimic BPPV, a condition termed central positional vertigo (CPV). Indeed, two retrospective studies revealed that 11–12% of all patients with PN had central pathologies, such as vertebrobasilar stroke or posterior fossa neoplasms [3, 4]. The risk of misdiagnosis appears to depend on the observed BPPV pattern, being highest when the presenting symptoms are consistent with (apogeotropic) lateral canal BPPV [5, 6]. Here we present a patient with cerebellar metastases who showed transient response to liberation maneuvers and a shift from apogeotropic to geotropic PN, ultimately resembling an apogeotropic variant of lateral-canal BPPV. We discuss potential pathomechanisms to explain these clinical findings.

## Case Description

**Fig. 1:**
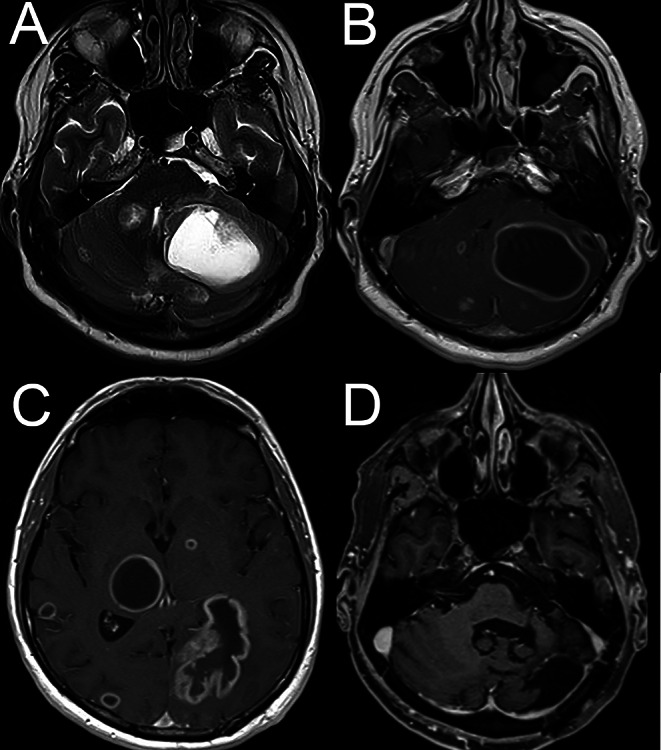
On magnetic resonance imaging (MRI) axial T2-weighted (panel **A**) and axial T1 post contrast (panel **B**) images demonstrate multiple cystic cerebellar space-occupying lesions compressing the fourth ventricle. Additionally, multiple supratentorial cystic mass lesions were depicted on MRI (panel **C**, axial T1 post contrast sequence). At follow-up after 9 months (panel **D**) a status post left-sided sub-occipital craniotomy and resection of the large left-cerebellar cystic mass lesion can be seen. (Source: Department of Radiology, Cantonal Hospital of Baden, Baden, Switzerland)

A 44-year-old previously healthy man presented to the emergency department with a 10-day of sudden-onset brief (lasting 1–2 min) PV with nausea/vomiting in right-ear-down (RED) and left-ear-down (LED) position and gait-imbalance. On initial examination, he demonstrated a left-beating horizontal nystagmus in supine position and showed marked sway when walking independently, referring to an acute imbalance syndrome. The neurologic examination was otherwise unremarkable, i.e., no spontaneous nystagmus in upright position, no gaze-evoked nystagmus, no skew-deviation, no head-shaking nystagmus. Hallpike-Dix testing did not provoke any PV or PN. Positioning maneuvers in the plane of the lateral canals (supine-roll test, SRT) on a manual rotatory chair (Rotundum, Balcare, Switzerland) performed the next day revealed a mild horizontal apogeotropic persistent PN and PV in RED-position and moderate-to-severe horizontal apogeotropic PN and PV in LED-position without any crescendo-decrescendo pattern and without nystagmus in supine position. A diagnosis of an apogeotropic variant of right lateral canal BPPV was made and a 360°-Barbecue repositioning maneuver to the left was performed twice, followed by a Gufoni-maneuver (nose up) (Table 1), with no PV/PN on subsequent SRT. At follow-up the next day, however, the patient again presented with a horizontal apogeotropic persistent PN/PV. After one 360° BBQ-maneuver to the left a geotropic horizontal PN with similar intensity in LED and RED position was noted in SRT. This was interpreted as a now geotropic-variant of right lateral-canal canalolithiasis and was treated with a 360° BBQ-maneuver to the right twice, again with no PV/PN on subsequent SRT. At 2nd follow-up three days later, the patient again presented with a apogeotropic PN and PV of moderate intensity. Again, after a 360° BBQ-maneuver to the left a shift to a geotropic-variant was observed and treated with a 360°-Barbecue maneuver to the right. On immediately subsequent SRT no PV/PN could be observed. Due to the recurrent findings and persistent moderate gait imbalance, a brain-MRI was obtained, demonstrating multiple intracranial, mainly cystic lesions with compression of the 4th -ventricle, the brainstem and the left cerebellar hemisphere (Fig. 1A-D). Emergency neurosurgical resection of the left-cerebellar mass revealed a diagnosis of metastases of an until then undiagnosed TTF1 (thyroid-transcription-factor 1) positive adenocarcinoma of the lung.

**Table 1:** Sequence of clinical findings and treatment maneuvers applied

Visit	Findings	Interpretation	Reposition maneuver(s) on the turntable	Findings on subsequent evaluation
Day 2	90° SRT: • Persistent apogeotropic horizontal nystagmus, LED > > RED • Intense vertigo	Apogeotropic variant of right lateral canal BPPV	• 2 × 360° BBQ-maneuver to the left • 1x Gufoni-maneuver to the right (nose up)	No nystagmus or vertigo on 90° SRT
Day 3	90° SRT: • Persistent apogeotropic horizontal nystagmus, LED > > RED • Moderate vertigo	Persistent apogeotropic variant of right lateral canal BPPV	• 1 × 360° BBQ-maneuver to the left	Geotropic horizontal nystagmus (LED = RED) on 90° supine roll test: → Interpretation: geotropic variant of right lateral canal BPPV → Treatment: 2 × 360° BBQ-maneuver to the right → Re-assessment after treatment: no nystagmus or vertigo on 90° SRT
Day 6	90° SRT: • Persistent apogeotropic horizontal nystagmus, LED > > RED • Moderate vertigo	Persistent apogeotropic variant of right-lateral canal BPPV	• 1 × 360° BBQ-maneuver to the left	Geotropic horizontal nystagmus (RED > LED) on 90° supine roll → Interpretation: geotropic variant of right lateral canal BPPV → Treatment: 1 × 360° BBQ-maneuver to the right → Re-assessment after treatment: no nystagmus or vertigo on 90° SRT
9 months	90° SRT: • Persistent apogeotropic horizontal nystagmus, LED > > RED • No vertigo	Central paroxysmal positional vertigo	• None	

Abbreviations: BBQ = Barbecue; LED = left-ear down; RED = right ear down; SRT = supine roll test

Combined whole-brain radiation and chemotherapy with an EGRF-inhibitor (Osimertinib) was initiated. On follow-up after 9 months the patient demonstrated moderate gait imbalance and sway on Romberg test, and horizontal saccades were hypermetric. On SRT, a persistent apogeotropic PN without accompanying nausea/vertigo was observed (Table 1). However, further tumor progression was noted on oncologic follow-up, resulting in the patient’s death 2 years after diagnosis.

## Discussion

This patient presented several characteristic features of lateral-canal BPPV, including a suitable history with brief position-dependent dizzy spells and nausea, an apogeotropic PN that was stronger on one side in SRT and a (transient) response to liberation maneuvers. At the same time, he did not show any focal neurologic/oculomotor signs suggestive of an acute cerebellar/brainstem lesion except moderate gait imbalance, which may also be observed in acute BPPV cases. Thus, this case underlines the importance of follow-up consults even after seemingly successful liberation of BPPV, especially for suspected apogeotropic lateral-canal BPPV, which is one of the least frequent BPPV-variants, but at the same time the most frequently seen pattern of central PN (CPN) [6, 7]. Furthermore, this case emphasizes the possibility of changing PN-patterns in central cases as well, further complicating the distinction between peripheral and central pathologies. Structural lesions resulting in central PV (CPV) and CPN are typically located around the 4th -ventricle and may involve brainstem areas such as the lateral medulla or the nucleus prepositus hypoglossi, cerebellar structures (including the caudal vermis (nodulus, uvula) and the tonsils [8]), or the cerebellar peduncles [9, 10].

CPN has been explained by an abnormal central processing and integration of SCC-related signals and otolith-related signals [11] in addition to a prolonged velocity-storage due to vestibulo-cerebellar dysfunction [7, 8, 12]. Thus, calculated head- and eye-position signals will be erroneous. Subsequently, seemingly compensatory eye movements such as a persistent apogeotropic PN are elicited during SRT [7, 8, 12]. Perceptually, this is usually accompanied by paroxysmal or persistent PV. Misdiagnosis is frequent: in a patient-cohort with suspected lateral-canal BPPV, diagnosis was revised to CPN in 11% of cases after an extensive diagnostic workup [6].

## The Diagnostic Value of the PN-Pattern Observed

A torsional-geotropic PN on Hallpike-Dix maneuver (HDM) has rarely been linked to central causes, and at the same time is the most frequent BPPV-variant [2]. However, other nystagmus patterns may be seen in HDM that more likely point to a central cause (Table 2). This includes a positional downbeat nystagmus, which may be rarely seen also in anterior-canal BPPV [13] and also in the apogetropic variant of posterior-canal BPPV. In cases presenting with an apogeotropic/geotropic PN on SRT, this may be due to lateral-canal BPPV or due to central causes (Table 2). While the apogeotropic variant of lateral-canal BPPV is less frequently observed than the geotropic variant, an apogeotropic PN is the most common CPN-pattern [6]. Thus, risk of misdiagnosis is much higher than e.g. for posterior-canal BPPV. Other clinical features such as nystagmus dynamics, latency and duration of episodes are much less reliably and should be interpreted with caution (Table 2).

**Table 2:** The range of nystagmus patterns in different provocation maneuvers in BPPV and CPV [5, 9, 10, 14]

Provocation maneuver	Nystagmus patterns in BPPV	Nystagmus patterns in CPV
Hallpike-Dix maneuver (HDM) for assessing the posterior (anterior) semicircular canals	Torsional-geotropic nystagmus in case of posterior-canal BPPV with - Crescendo-decrescendo pattern - Latency of few seconds - Duration usually 5–15 s Vertical (downbeating) nystagmus (with/without apogeotropic torsional nystagmus in case of rare anterior-canal BPPV variant)	Downbeat nystagmus - paroxysmal (duration usually 10–15 s) or persistent (> 60 s duration) Very rarely horizontal (geotropic or apogeotropic), torsional or vertical (upbeating) nystagmus
90° Supine Roll Test (SRT) for assessing the lateral semicircular canals	Horizontal geotropic or (less frequently) apogetropic nystagmus - Latency very short or lacking - Duration very variable, may be persistent	Horizontal apogeotropic or (much less frequent) geotropic nystagmus - Sometimes only observed during SRT to one side - Latency short or absent - Duration very variable (paroxysmal < persistent) Very rarely vertical (upbeating or downbeating) nystagmus
Straight head hanging (SHH)	Torsional and upbeating nystagmus for posterior-canal BPPV	Vertical downbeating nystagmus

Abbreviations: BPPV = benign paroxysmal positional vertigo; CPV = central positional vertigo

If treatment-response to liberation maneuvers is lacking or transient only and/or if the nystagmus pattern observed during the provocation maneuver is atypical for BPPV, a CPN should be suspected and brain-MRI including contrast-enhanced sequences should be ordered. In cases with no evidence for structural lesions and no vestibular migraine, lumbar puncture should be performed to search for (autoimmune) inflammatory causes.

## Potential Pathomechanisms for the Observed Shifts in PN-Patterns

Unique to the case presented here is the shift from apogeotropic to geotropic PN on provocation maneuvers and finally transiently resolved PN/PV following sequential liberation maneuvers. This observation would usually be considered reflecting treatment response and thus being suggestive of a benign, peripheral origin and confirming the BPPV-diagnosis. In contrast, lack of response to liberation maneuvers is considered a defining feature of CPN [10, 11, 14]. Obviously, this assumption does not hold true in the case presented here. Furthermore, it raises the question how these shifting PN-patterns may be explained by the structural changes identified and the positional maneuvers applied.

In a proposed model of apogeotropic CPN, it was assumed that the position-induced nystagmus is due to the error in head-centered gravity estimation, as this was the only variable that changed between LED and RED position [7]. Previously, it has been shown that the nodulus and uvula play a critical role in the processing of gravity estimation. This has been demonstrated in animal experiments with complete nodulo-uvulectomy showing an increase in the time-constant of post-rotatory nystagmus and optokinetic after-nystagmus in the supine or ear-down position from 8 to 12 s to 50 s [15] and in humans (demonstrating reduced tilt-suppression of post-rotatory nystagmus in patients with isolated stroke of the uvula and nodulus [16]). Muscimol injection into the nodulus and the uvula in the monkey also produced an apogeotropic PN with a linear relationship between the nystagmus intensity and the gravitational orientation of the head [17]. Furthermore, the eminent role of the nodulus in the processing of gravity estimation was demonstrated in a single human patient with heminodular stroke, presenting with a transient loss of otolith-perceptual integration [18]. Likely, the modulatory effect of head-position relative to gravity on eye-position is mediated through the velocity-storage mechanism (VSM) [19]. By reinterpreting the VSM as an internal estimator of angular velocity and gravitational acceleration [20] and by assuming a lesion that affects the pathway between the gravity estimator and the rotational feedback loop, a persistent horizontal PN could be modeled [7]. Notably, the proposed mechanism implies that, depending on the direction of bias, persistent horizontal PN can manifest as either apogeotropic or geotropic.

Potentially, bringing our patient into different orientations relative to gravity by various repositioning maneuvers may have resulted in a changing compression of cerebellar/brainstem structures. Thereby the large left-hemispheric cerebellar cystic mass lesion with a medially-positioned solid component and a laterally-placed large cystic component (Fig. 1) may have played an important role. A 360-degree rotation might have induced fluid motion within the mass, requiring time to stabilize. Consequently, this could exert different effects on the nodulus and uvula during such a transient phase in response to 360-degree rotations before a steady-state phase is reached again. Thus, the position-dependent increasing and decreasing compression of cerebellar/brainstem structures and partial obstruction of the 4th -ventricle may have resulted in different pathological effects, which caused varying biases in gravity estimation, ultimately leading to apogeotropic horizontal PN in the steady-state (i.e., after adaptation is reached), while inducing geotropic horizontal PN or even eliminating PN during the transient period in response to a 360-degree Barbecue rotation. Such a mechanism would be analogous to other posture-related symptoms described in the literature such as orthostatic headaches in patients with cerebellar hemorrhage [21] and infratentorial brain tumors [22].

Alternatively, periodic neuronal discharges could potentially explain shifting PN-patterns. Specifically, periodically changing nystagmus-patterns have been described in single patients with cerebellar dysfunction, either due acute hemorrhage of the cerebellar nodulus/uvula [23] or due to a paraneoplastic cerebellar syndrome [24]. However, with the change in PN beating-direction triggered by specific liberation maneuvers, this makes a shift in direction due to periodic neuronal discharges rather unlikely.

Furthermore, we cannot fully exclude a combined BPPV-CPV pattern to explain the changing PN-beating directions. If the apogeotropic PN actually were due to a right lateral-canal BPPV, successful liberation maneuvers could have unmasked the central (geotropic) pattern. However, with a residual apogeotropic horizontal PN observed post-resection, this pattern rather suggests that the apogeotropic PN is of central origin and not due to a lateral-canal BPPV.

Modeling of CPN, has not taken into account the effect of liberation maneuvers on CPN patterns [7]. Thus, future studies are needed to address this open question.

## Conclusions

Central causes must be suspected when progressive, treatment-resistant (paroxysmal) PN and PV in combination with other neurological deficits occur or if nystagmus patterns are atypical during provocation maneuvers. Furthermore, short-lasting responses to liberation maneuvers may be seen in CPN also, mimicking the response-pattern expected in BPPV-cases. Thus, response to treatment must be validated on a follow-up consultation. Varying pressure by a cystic cerebellar mass lesion on the nodulus and uvula that varies with changing head-position relative to gravity might potentially explain adaptational changes in horizontal PN-direction and subsequent cessation.

## Data Availability

The data that support the findings of this case study are available from the corresponding author upon reasonable request.
